# Extramitochondrial Ca^2+^ in the Nanomolar Range Regulates Glutamate-Dependent Oxidative Phosphorylation on Demand

**DOI:** 10.1371/journal.pone.0008181

**Published:** 2009-12-09

**Authors:** Frank Norbert Gellerich, Zemfira Gizatullina, Odeta Arandarcikaite, Doreen Jerzembek, Stefan Vielhaber, Enn Seppet, Frank Striggow

**Affiliations:** 1 KeyNeurotek Pharmaceuticals AG, ZENIT Technology Park, Magdeburg, Germany; 2 Department of Neurology, Otto von Guericke University Magdeburg, Magdeburg, Germany; 3 Institute for Biomedical Research, Kaunas University of Medicine, Kaunas, Lithuania; 4 Department of Pathophysiology, Centre of Molecular and Clinical Medicine, University of Tartu, Tartu, Estonia; National Institutes of Health, United States of America

## Abstract

We present unexpected and novel results revealing that glutamate-dependent oxidative phosphorylation (OXPHOS) of brain mitochondria is exclusively and efficiently activated by extramitochondrial Ca^2+^ in physiological concentration ranges (S_0.5_ = 360 nM Ca^2+^). This regulation was not affected by RR, an inhibitor of the mitochondrial Ca^2+^ uniporter. Active respiration is regulated by glutamate supply to mitochondria via aralar, a mitochondrial glutamate/aspartate carrier with regulatory Ca^2+^-binding sites in the mitochondrial intermembrane space providing full access to cytosolic Ca^2+^. At micromolar concentrations, Ca^2+^ can also enter the intramitochondrial matrix and activate specific dehydrogenases. However, the latter mechanism is less efficient than extramitochondrial Ca^2+^ regulation of respiration/OXPHOS via aralar. These results imply a new mode of glutamate-dependent OXPHOS regulation as a demand-driven regulation of mitochondrial function. This regulation involves the mitochondrial glutamate/aspartate carrier aralar which controls mitochondrial substrate supply according to the level of extramitochondrial Ca^2+^.

## Introduction

It has been assumed that ADP formed by ATP-consuming enzymes activates OXPHOS [1]. However, cytosolic ADP of the heart muscle is only insignificantly increased *in vivo* during elevated work loads [2], [3]. Therefore, two hypotheses have been proposed, (i) the dynamic compartmentation of ADP, assuming that necessary ADP augmentations occur exclusively within the mitochondrial intermembrane space [4], [5] and (ii) the stimulation of OXPHOS due to Ca^2+^ influx into the mitochondrial matrix via Ca^2+^ uniporter, followed by the activation of distinct intramitochondrial dehydrogenases [6], [7]. Some authors also assume a Ca^2+^ stimulation of F_0_F_1_-ATP synthase [8], [9]. However, both scenarios comply only partially with the *in vivo* findings outlined above [10].

Recent data suggest that the activity of the malate aspartate shuttle (MAS), including glutamate/aspartate carriers as aralar, is activated by extramitochondrial Ca^2+^ (S_0.5_ = 324 nM) [11]–[13]. The N-terminal regulatory Ca^2+^-binding site of aralar is located within the mitochondrial intermembrane space [11]–[13] where it can interact with Ca^2+^ passing through porin pores of the outer membrane. Aralar supplies OXPHOS with glutamate, a key mitochondrial substrate. In this study, we addressed the question whether OXPHOS can be directly activated by extramitochondrial Ca^2+^ and if so, whether aralar is involved in this regulation.

## Results

**Figure 1:**
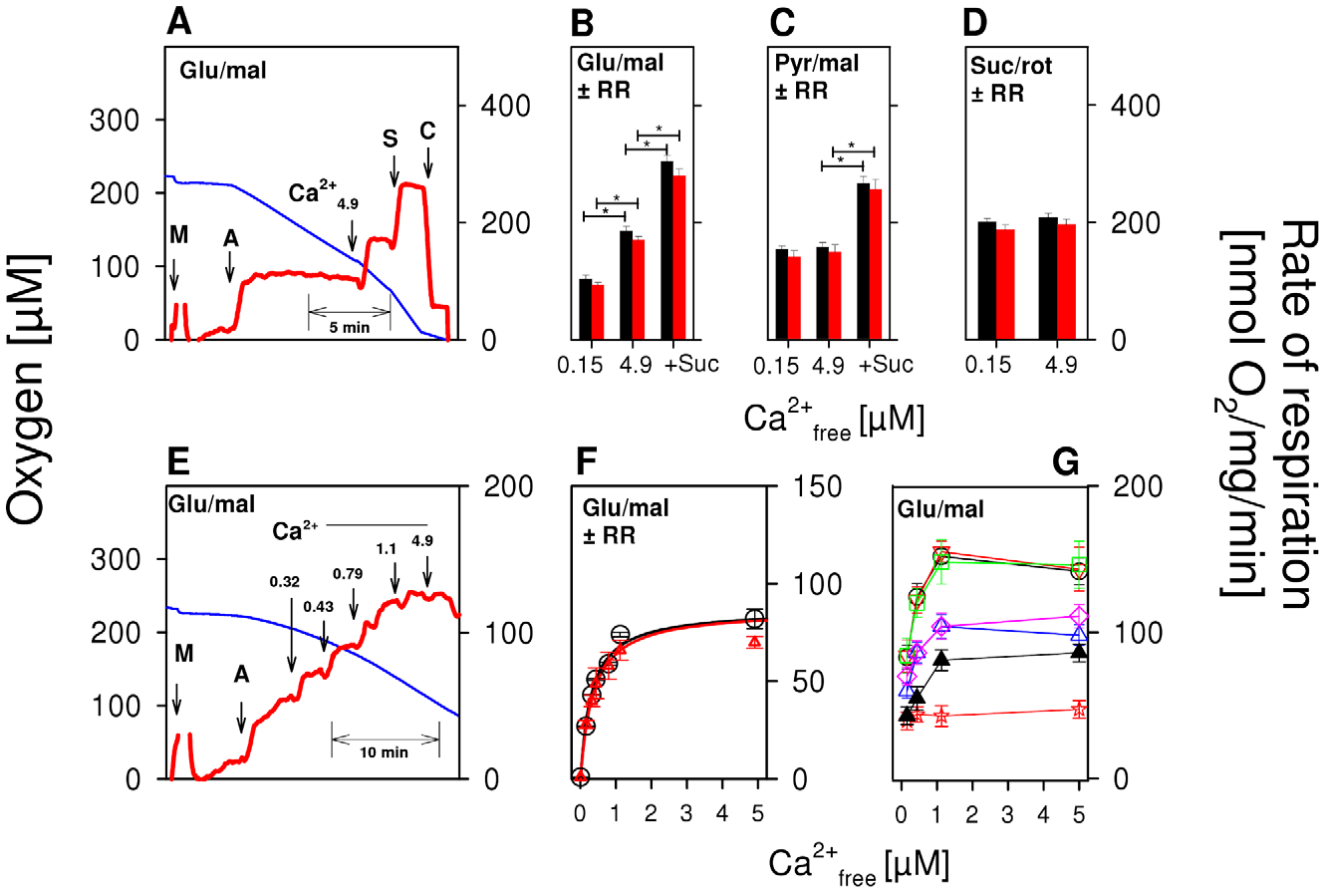
Exclusive activation of glutamate-dependent state 3 respiration of brain mitochondria by extramitochondrial Ca^2+^ in the nanomolar range. (A,E) Respirograms of rat brain mitochondria were obtained by high-resolution respirometry. (A) Isolated rat brain mitochondria were incubated in EGTA medium (Ca^2+^_free_ = 0.15 µM) in the presence of 10 mM glutamate and 2 mM malate as substrates. Additions: M, 0.06 mg/ml brain mitochondria, A, 2.5 mM ADP to activate the phosphorylation-related respiration (state 3); Ca^2+^_4,9_, 4.9 µM Ca^2+^_free_; S, 10 mM succinate as substrate of respiratory chain complex II; C, 5 µM carboxyatractyloside to block the adenine nucleotide translocase. Blue lines indicate the oxygen concentration and red lines represent respiration rates (nmol O_2_/mg mitochondrial protein/min). (B) Means of state 3 respiration±S.E. as measured in experiments shown in A without (black columns, n = 6) or with 250 nM RR, an inhibitor of mitochondrial Ca^2+^ uptake (red columns, n = 6). First group of columns, state 3 at Ca^2+^_free_ = 0.15 µM. Second group, state 3 with Ca^2+^_free_ = 4.9 µM. Third group, state 3 with Ca^2+^_free_ = 4.9 µM in the additional presence of 10 µM succinate. *, p<0.05. (C) As B, but derived from experiments with 10 mM pyruvate + 2 mM malate as substrates. *, p<0.05. (D) As B, but derived from experiments with 10 mM succinate + 2 µM rotenone as substrate. (E) Ca^2+^ titration of state 3_glu/mal_ by stepwise increase of Ca^2+^ as indicated either without (E,F) or with (F) 250 nM RR. (F) Incremental accretions of Ca^2+^-induced state 3_glu/mal_ were plotted against the fluorimetrically measured Ca^2+^ activity (Fig. 1F), allowing the calculation of the half-activation constant (S_0.5_) and the maximum velocity (V_max_) using the SigmaPlot kinetic module as given in the text. (G) Rates of state 3_glu/mal_ respiration obtained by Ca^2+^ titrations under various conditions. (○) Control mitochondria were investigated as in Fig. 1E. (□) As (○), but in the additional presence of 10% dextran 20. (▿) As (○), but in the additional presence of 1 mM CsA. (▵) as (○), but mitochondria isolated without digitonin were used. (◊) as (○), but mitoplasts were used. (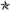) as (○), but mitochondria were uncoupled by 50 nM FCCP from the beginning of experiments, and then Ca^2+^ titration was performed. (▴) as (○), but Ca^2+^ was adjusted at the beginning of experiments as indicated. Thereafter, 100 µM ADP was added, causing short transitions between the active and resting states of respiration. After reaching state 4 respiration, FCCP titrations were performed to uncouple respiration and ATP generation. Maximum respiration rates were obtained at 60 or 80 nM FCCP and were plotted against the Ca^2+^_free_ value for the respective incubation. Data are means±S.E. of 4 independent experiments.

First, we investigated the influence of Ca^2+^ on OXPHOS of isolated rat brain mitochondria in a medium containing 150 nM free Ca^2+^ (Ca^2+^_free_), corresponding to basal levels of cytosolic Ca^2+^ under physiological conditions [14]. ADP was added so as to fully activate phosphorylation-related respiration (state 3). Using glutamate/malate as substrate, a relatively low state 3_glu/mal_ was obtained (Fig. 1A,B). However, state 3_glu/mal_ nearly doubled immediately after a pulse addition of 4.9 µM Ca^2+^_free_ (Fig. 1A,B). This Ca^2+^ activation was not limited by the mitochondrial capacity of OXPHOS, but rather was due to its efficacy in metabolizing glutamate, as succinate conspicuously enhanced respiration above the level of state 3_glu/mal_. With pyruvate/malate (Fig. 1C), state 3_pyr/mal_ significantly exceeded state 3_glu/mal_ (Fig. 1A,B). However, added Ca^2+^ did not augment state 3_pyr/mal_, whereas added succinate did (Fig. 1C). Fig. 1D demonstrates that there was also no Ca^2+^ effect on complex II-dependent state 3_suc_ with succinate/rotenone. Overall, these results show that Ca^2+^ activation of OXPHOS in isolated brain mitochondria is a glutamate-specific phenomenon. The next series of experiments revealed that RR, an inhibitor of the mitochondrial Ca^2+^ uniporter [15], is not able to modulate Ca^2+^ effects on state 3 with any substrate (Fig. 1B–D). We performed these experiments in the presence of relatively low RR concentrations (250 nM) in order to avoid possible unspecific RR effects. Nevertheless, even in the presence of up to 5 µM RR, extramitochondrial Ca^2+^-induced stimulation of state 3_glu/mal_ was detectable (Data not shown).

Next, we investigated the kinetics of Ca^2+^ activation (Fig. 1E,F). Ca^2+^ was increased in steps. Increments of Ca^2+^-induced state 3_glu/mal_ were plotted against fluorimetrically measured Ca^2+^ (Fig. 1F) in order to determine the half-activation constant (S_0.5_) and the extent of Ca^2+^ stimulation (S_0.5_ = 356±39 nM Ca^2+^_free_, V_max_ = 86±5 nmol O_2_/mg/min). Neither parameter was affected by RR (S_0.5_ = 306±35 nM Ca^2+^_free_, V_max_ = 88±8 nmol O_2_/mg/min). Thus, Ca^2+^ influx into the mitochondrial matrix appears not to be required for state 3_glu/mal_ stimulation and, hence, Ca^2+^ activation must be an extramitochondrial effect.

To exclude furthermore artificial Ca^2+^ effects due to potential interactions of digitonin with mitochondrial membranes, we also varied the digitonin concentration used during the preparation of mitochondria to permeabilize synaptosomal membranes. Omitting digitonin did not cause any significant changes in the extent of extramitochondrial Ca^2+^ activation of state 3_glu/mal_ (Fig. 1G) compared with control mitochondria prepared with digitonin (Fig. 1G). Thus, digitonin-related artifacts can be excluded. It should be noted that in the absence of digitonin, synaptosomal mitochondria remained inaccessible, and therefore respiratory rates were significantly decreased in digitonin-free experiments (Fig. 1G). On the other hand, large additions of digitonin (1.2 mg digitonin/mg mitochondrial protein) led to a removal of mitochondrial outer membranes and the generation of mitoplasts [16], [17]. Consequently, the accessibility of mitochondrial Ca^2+^-binding sites, originally located within the inner membrane space, to Ca^2+^ was facilitated but no changes of Ca^2+^ activation were detectable (Fig. 1G). As previously observed in heart mitoplasts [17], we also registered lower respiratory rates compared with control mitochondria; this was probably due to unspecific side effects of digitonin on mitoplasts. This finding suggests that Ca^2+^ diffusion through porin pores of the mitochondrial outer membrane does not limit its interaction with mitochondrial Ca^2+^-binding sites exposed into the inner membrane space and thus, does not compromise extramitochondrial Ca^2+^ regulation of glutamate/malate-dependent respiration and OXPHOS. Another experimental setup was used to obtain support for this interpretation. In intact cells, the colloid osmotic pressure increases the diffusion resistance of the mitochondrial outer membrane against metabolites passing the porin pores [18]. We therefore simulated the intracellular oncotic pressure by addition of 10% dextran [18], but again observed a similar extramitochondrial Ca^2+^ stimulation of state 3_glu/mal_ respiration and OXPHOS (Fig. 1G). Moreover, the addition of 2 µM cyclosporine A (CsA), an inhibitor of the mitochondrial permeability pore (PTP)[19], did not affect the extramitochondrial Ca^2+^ regulation of brain mitochondria (Fig. 1G), suggesting that PTP is not involved in the phenomenon of extramitochondrial Ca^2+^ regulation of state 3_glu/mal_ and OXPHOS.

We then investigated the influence of mitochondrial uncoupling on Ca^2+^ stimulation of OXPHOS. Since aralar is an electrogenic carrier [20], glutamate transport into mitochondria requires a sufficiently high mitochondrial membrane potential. Accordingly, mitochondria uncoupled by 50 nM FCCP at the beginning of the experiment could not be activated by the following Ca^2+^ titration, owing to the dissipation of membrane potential (Fig. 1G). In a second approach, different Ca^2+^_free_ concentrations were initially adjusted followed by FCCP titration of the nonphosphorylating respiration (state 4). This application scheme resulted in enhanced maximum rates of uncoupled respiration in a Ca^2+^ -dependent manner (Fig. 1G). However, since FCCP also caused an incomplete dissipation of mitochondrial membrane potentials, maximum rates of uncoupled respiration were lower than in control experiments without FCCP (Fig. 1G). Obviously, cytosolic Ca^2+^ can modulate glutamate transport rate via aralar but is not able to adjust the thermodynamic conditions necessary for glutamate uptake.

Therefore, several lines of experimental evidence clearly support the assumption that extramitochondrial Ca^2+^ regulation of glutamate-dependent OXPHOS is a physiologically relevant phenomenon, rather than being an experimental artifact.

**Figure 2:**
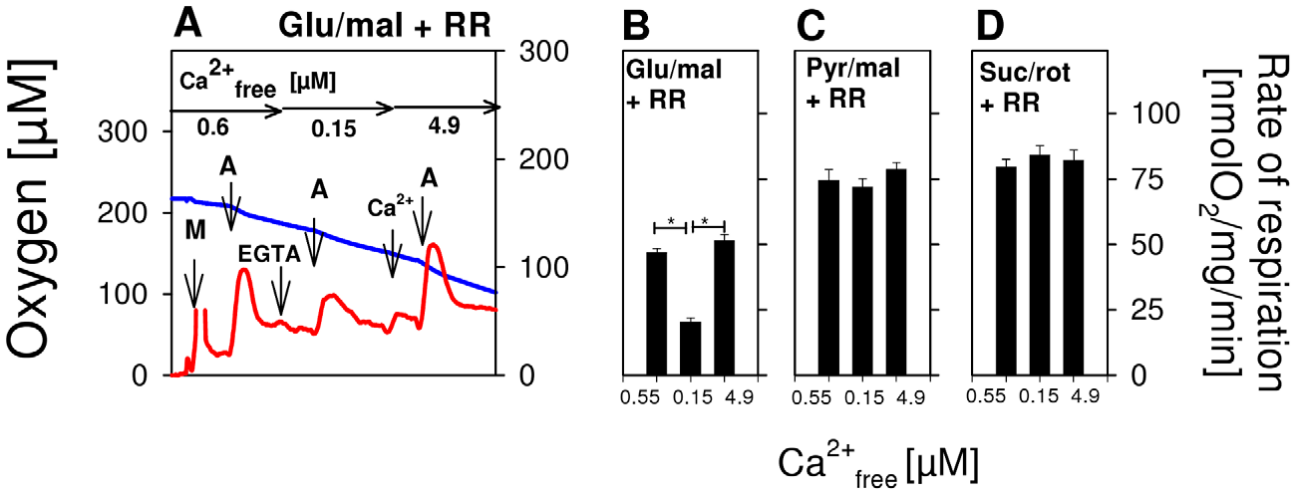
Exclusive and reversible activation of glutamate-dependent respiration by extramitochondrial Ca^2+^ at low levels of ADP. (A) Isolated rat brain mitochondria (0.06 mg/ml) were incubated in EGTA-free medium (0.6 µM Ca^2+^_free_) with 10 mM glutamate and 2 mM malate as substrates, but in the presence of 250 nM RR. Additions: M, 0.06 mg/ml rat brain mitochondria; A, 150 µM ADP; EGTA, 100 µM EGTA (0.15 µM Ca^2+^_free_); Ca^2+^_4.9_, 4.9 µM Ca^2+^_free_. Horizontal arrows indicate the actual Ca^2+^_free_ concentration. (B–D). Means of phosphorylating respiration±S.E. were calculated as stationary state 3 respiration rate minus state 4 respiration rate from measurements as shown for glutamate and malate in A at defined extramitochondrial Ca^2+^. Different substrates were used as indicated. *P<0.01.

In intact cells, mitochondria are not exposed to such high ADP concentrations as applied here (Fig. 1). In order to address this issue in more detail, we investigated whether Ca^2+^ can also stimulate glutamate-dependent respiration at physiological ADP levels and, if so, whether this stimulation by Ca^2+^ is a reversible phenomenon. These measurements were started in EGTA-free medium (Ca^2+^_free_ ∼0.6 µM) containing RR. With glutamate/malate as substrates, ADP (150 µM) caused an intermediate activation of phosphorylating respiration with a maximum rate of 50 nmol O_2_/mg/min (calculated by subtracting state 4 from state 3 respiration; Fig. 2A,B). By the addition of 100 µM EGTA, Ca^2+^_free_ was then lowered to ∼150 nM, which was less than half the value of S_0.5_ = 360 nM for Ca^2+^ activation of state 3_glu/mal_ (Fig. 1F). Under these conditions, the rate of ADP-induced respiration was significantly reduced compared to experiments in the presence of higher Ca^2+^_free_ levels (Fig. 2A,B). Increasing of Ca^2+^_free_ up to 4.9 µM again markedly accelerated state 3_glu/mal_ and OXPHOS, demonstrating perfect reversibility of extramitochondrial Ca^2+^ regulation. In contrast, similar Ca^2+^ changes did not affect OXPHOS rates using pyruvate/malate (Fig. 2C) or succinate/rotenone as substrates (Fig. 2D).

**Figure 3:**
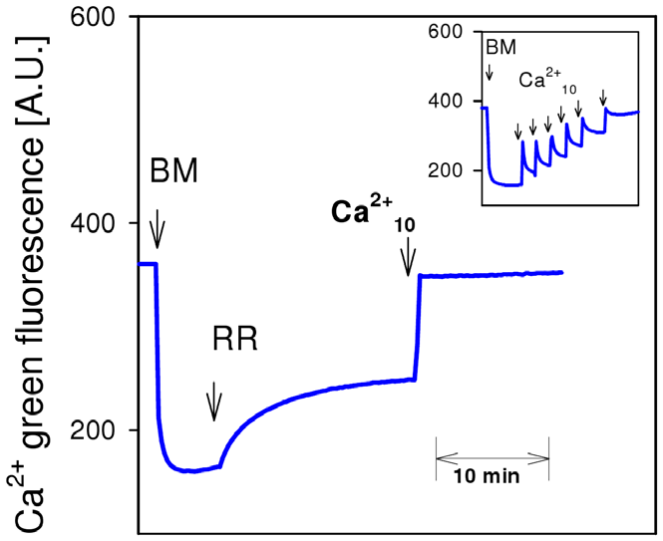
Brain mitochondria do not accumulate, but rather lose, Ca^2+^ in the presence of ruthenium red. Fluorimetric measurement of extramitochondrial Ca^2+^ with Ca^2+^green. Brain mitochondria were incubated in EGTA-free medium with 10 mM glutamate and 2 mM malate. Additions: BM, 0.25 mg/ml brain mitochondria; RR, 250 nM ruthenium red (RR); Ca^2+^_10_, 10 µM Ca^2+^_free_, Insertion: Control experiment without RR demonstrating normal Ca^2+^ accumulation of brain mitochondria after repeated Ca^2+^ additions.

In order to find out whether 250 nM RR is able to inhibit mitochondrial Ca^2+^ uptake via Ca^2+^ uniporter, we performed another experiment using fluorimetric Ca^2+^ measurements in EGTA-free medium. It is well known that repeated Ca^2+^ additions lead to a sequential and reversible increase of Ca Green fluorescence due to respective changes in extramitochondrial Ca^2+^ (Fig. 3, insertion). In line with previous reports, addition of RR to isolated brain mitochondria induced a significant increase in extramitochondrial Ca^2+^ which was caused by a net Ca^2+^ release from mitochondria (Fig. 3A) [21]. The subsequent addition of 10 µM Ca^2+^ induced a sustained increase of Ca^2+^ Green fluorescence, confirming effective inhibition of the Ca^2+^ uniporter by RR, which is also in accordance with earlier reports [21].

In the next series of experiments, we determined the K_M_ of mitochondrial Ca^2+^ uptake via Ca^2+^ uniporter under conditions used in here. The estimated K_M_ of 3.7±0.9 µM Ca^2+^_ free_ exceeds the S_0.5_ of Ca^2+^-activated respiration (360 nM Ca^2+^_ free_, Fig. 1F) about 10-fold. Such a big difference between K_M_ and S_0.5_ suggests that mitochondrial Ca^2+^ accumulation cannot take place as long as extramitochondrial Ca^2+^ remains within the nM concentration range. To verify this important conclusion, Ca^2+^_free_ was monitored directly with Fura-2 under conditions otherwise equivalent to those in respirometric experiments with glutamate/malate and 100 µM EGTA-medium (not shown). At Ca^2+^_free_ levels up to 1.2 µM, mitochondrial Ca^2+^ accumulation was not detectable. Only after further Ca^2+^ additions did mitochondrial Ca^2+^ uptake become visible (not shown).

## Discussion

It is widely believed that increased cytosolic Ca^2+^ exerts a parallel activation of extramitochondrial ATPases and OXPHOS, thereby balancing exactly ATP consumption and production without major changes in ADP concentration [2], [3], [6], [7], [8], [10]. Ca^2+^ transport into the mitochondrial matrix and subsequent activation of distinct intramitochondrial dehydrogenases [2], [3], [6], [7], [22], [23] and F_0_F_1_ATPase [8], [9] are assumed to constitute the regulatory mechanism of mitochondrial respiration and OXPHOS. However, an exclusive activation of OXPHOS by intramitochondrial Ca^2+^ is questionable in the light of following arguments. (i) Computer modeling of intramitochondrial Ca^2+^ activation of OXPHOS was unable to simulate the OXPHOS activation in response to physiological changes of work load *in vivo* [10]. (ii) The low affinity of the mitochondrial Ca^2+^ uniporter to Ca^2+^_free_ (K_M_ = 3.7±0.9 µM) should not allow an effective increase in intramitochondrial Ca^2+^ effectively under conditions of only slightly elevated Ca^2+^. Therefore, detectable mitochondrial Ca^2+^ uptake at nanomolar Ca^2+^ levels was explained by spatial heterogeneity of cytosolic Ca^2+^ concentration [24] and/or by a spermine-induced increase in the uniporter's affinity for extramitochondrial Ca^2+^ [25], [26]. (iii) Moreover, the relative insensitivities of intramitochondrial dehydrogenases to Ca^2+^ (S_0.5_ = 0.4 - 13 µM Ca^2+^_free_) [22], [23] require significant higher Ca^2+^_free_ levels for their activation compared with extramitochondrial Ca^2+^ activation of state 3_glu/mal_ and OXPHOS. Thus, the function of mitochondrial Ca^2+^ uptake and accumulation appears rather to serve as reversible Ca^2+^ buffer, ensuring intracellular Ca^2+^ homeostasis, than to regulate state 3_glu/mal_ and OXPHOS [14].

This study reveals a novel mechanism of extramitochondrial Ca^2+^ activation of state 3_glu/mal_ and OXPHOS mediated by aralar. This finding is supported by several earlier observations. (i) RR inhibits cardiac function only slightly *in vivo* [27], [28], suggesting that mitochondrial Ca^2+^ uptake is not obligatory for stimulation of mitochondrial ATP production *in vivo*. (ii) In contrast, AOA, an inhibitor of MAS, attenuates the respiration of isolated synaptosomes [29] and suppresses the contractile function of the perfused, working heart [30], when glucose or lactate are oxidized. On the other hand, full contractile functionality can be observed if pyruvate is used in the presence of AOA [30].

Since pyruvate formation and aralar function are tightly interconnected in intact cells [11], extramitochondrial Ca^2+^, beside its regulation of MAS [11]-[13], also regulates pyruvate formation from glucose or lactate. Since pyruvate is the main substrate of brain mitochondria [31], extramitochondrial Ca^2+^ is able to adjust the supply of OXPHOS with its main substrates precisely and reversibly, like a physiological “gas pedal”, acting in response to distinct, Ca^2+^-mediated cellular demands.

Taken together, our results imply a new and consistent feature of OXPHOS regulation in brain mitochondria in which the mitochondrial glutamate/aspartate carrier aralar controls mitochondrial substrate supply and OXPHOS according to the extramitochondrial level of Ca^2+^.

## Materials and Methods

### Mitochondria

Brain mitochondria (containing synaptosomal and nonsynaptosomal fractions) were isolated from 3–4-month-old Wistar WU rats (Charles River Laboratories, Germany) according to the protocol by Kudin *et al*., which includes permeabilization of synaptosomes with digitonin [32]. Isolation and incubation media did not contain bovine serum albumin (BSA). Before final suspension, the mitochondrial Ca^2+^ content was routinely diminished by extraction with nitriloacetic acid using the method of Brandt *et al*. [33]. For some experiments shown in Fig. 1G, mitochondria were isolated without digitonin. These mitochondria were also used to prepare mitoplasts by short term incubation with 1.2 mg digitonin/mg mitochondrial protein similarly as described previously for heart mitoplasts [17]. All research and animal-care procedures were performed according to European guidelines.

### Respirometry

Mitochondrial respiration was measured with a Clark-type oxygen electrode by means of high-resolution respirometry [34], [35] using an OROBOROS oxygraph-2k (Oroboros, Innsbruck, Austria) at 30°C. Respiration of mitochondria (0.06 mg protein/ml) was measured in a medium containing 120 mM mannitol, 40 mM MOPS, 5 mM KH_2_PO_4_, 60 mM KCl, 5 mM MgCl_2_, and either 0 or 100 µM EGTA, pH 7.4. Ca^2+^_free_ concentrations in the various media were measured with Fura-2 as described below. EGTA-free medium contained 0.6 µM Ca^2+^_free_. 100 µM EGTA medium contained 0.15 µM Ca^2+^_free_.

### Ca^2+^ accumulation measurements

Ca^2+^ accumulation by isolated mitochondria (0.25 mg protein/ml) was monitored fluorimetrically in the presence of 0.5 µM Calcium Green-5N (Invitrogen) in a medium containing 120 mM mannitol, 40 mM MOPS, 5 mM KH_2_PO_4_ and 60 mM KCl. Measurements were performed in stirred and thermostatted (30°C) cells using a Carry Eclipse fluorimeter (Varian Deutschland GmbH) as described previously [36]. Excitation and emission wavelengths were set to 506 and 532 nm, respectively.

### Measurement of Ca^2+^_free_ in EGTA medium

Ca^2+^ in EGTA medium was measured fluorimetrically with Fura-2 (10 µM) as described previously [37]. The dissociation constant (K_d_) of the Ca^2+^-Fura-2 complex was determined experimentally under these conditions and was found to be 0.3 µM, which was similar to that found in a previous study [37].

### Protein determination

Mitochondrial protein concentrations were determined by the bicinchoninic acid assay [38], with BSA used as standard.
